# Immunotherapy Bridge 2020 and Melanoma Bridge 2020: meeting abstracts

**DOI:** 10.1186/s12967-020-02685-2

**Published:** 2021-03-24

**Oral Communications**

## 1. Cytokines and immune cell populations performance during nivolumab treatment. A subgroup analysis of NIVACTOR study

### Andrea Abbona^1^*, M. Paccagnella^1^, Nerina Denaro^2^, A. Falletta^1^, Danilo Galizia^3^, Lorena De Zarlo^3^, Erika Fiorino^5^, Loretta Gammaitoni^4^, Dario Sangiolo^5^, Marco Carlo Merlano^1^

#### ^1^Dipartimento di Oncologia, Azienda Ospedaliera St Croce e Carle, Cuneo, Italy; ^2^Medical Oncology, Azienda Ospedaliera St Croce e Carle, Cuneo, Italy; ^3^Medical Oncology, IRCCS Candiolo Institute, Turin, Italy; ^4^Medical Oncology, FPO-IRCCS Candiolo Institute, Turin, Italy; ^5^Department of Oncology, University of Turin, Turin, Italy

##### **Correspondence:** Andrea Abbona - abbona.andrea@gmail.com

*Journal of Translational Medicine* 2020; **19(Supp 1):** 1

**Background:** Recurrent or Metastatic Platinum-refractory Squamous Cell Carcinoma of the Head and Neck (R-M SCCHN) is a major clinical issue with 1 year survival rate of 20–30% and a median overall survival (OS) of 10 months [1, 2]. Nivolumab, anti-PD1 mAb, is approved for 2nd line R-M HNC, but only 15–20% of patients will benefit [3]. Therefore, there is an unmet need for robust predictive markers for patient selection.

**Patients and methods:** We analyzed changes of plasma circulating cytokines and immune cells during treatment at baseline (T0), at day 1 cycle 3 (T1), at day 1 cycle 7 (T2) and at disease progression (TPD). Cytokines’ concentrations were assessed using Simple Plexsystem (ProteinSimple). Immune cells from peripheral blood were analyzed by flow cytometry using FACS Cyan (Cyan ADP, Beckman Coulter) and analyzed with Summit Software. Principal component analysis (PCA) was performed to group patients with good or poor progression free survival (PFS) and OS using cut-off based on regression points of their extracted factors: group B (patients with factor 1 < 0.7 and factor 2 < 0.0) and remaining patients in group A.

P < 0.05 was considered to indicate significance.

**Results:** 18 patients were analyzed for both cytokine and immune cell populations. CCL-4 increased from T0 to T2 (P = 0.047) [Fig. 1]. Using PCA analysis we clustered patients in group B and group A on their T0 levels of IL-5, IL-6, CD3^+^CD8^+^LAG3^+^, CD3^+^CD8^+^PD1^+^LAG3^+^ and CD8^+^TEMRA [Fig. 2]. Then we performed Cox analysis using cut-off from regression points of their factors extracted with PCA observing a significant better PFS and OS in patients of group B (HR 0.185, P = 0.030 and HR 0.063, P = 0.010 respectively) [Fig. 3]. We also found that patients with increased levels of T_reg_ cells from T0 to T1 had better PFS (HR 0.098, P = 0.001) [Fig. 4]. Longitudinal analysis showed that TNF-α and IFN-γ increased from T0 to TPD (P = 0.049 and P = 0.035, respectively).

**Fig. 1:**
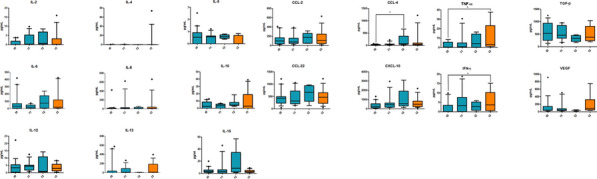
(Abstract 1) Distributions of 17 plasma cytokine levels during nivolumab treatment. Cytokines concentration was expressed in pg/mL. Data are shown as median with range. This Figure 1 belongs to Abstract 1

**Fig. 2:**
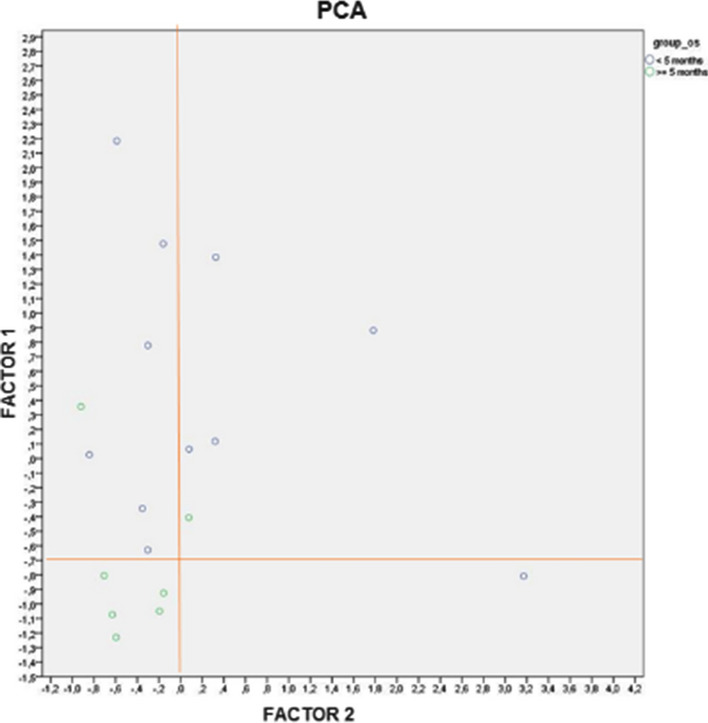
(Abstract 1) PCA analysis using T0 values of IL-5, IL-6, CD3^+^CD8^+^LAG3^+^, CD3^+^CD8^+^PD1^+^LAG3^+^ and CD8^+^TEMRA as parameters. Group B includes patients (n = 5) with factor 1 < 0.7 and factor 2 < 0; group A includes the other patients (n = 13). This Figure 2 belongs to Abstract 1

**Fig. 3:**
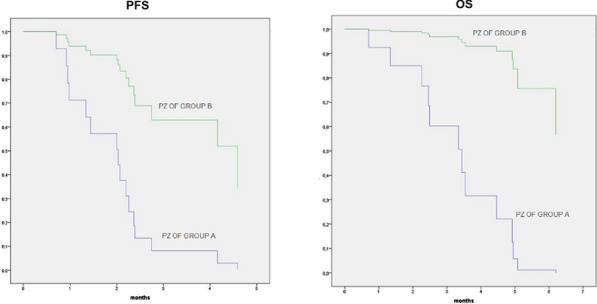
(Abstract 1) Cox analyses for PFS and OS of group A (blue line, n = 13) and group B (green line, n = 5) derived from PCA analysis. This Figure 3 belongs to Abstract 1

**Fig. 4:**
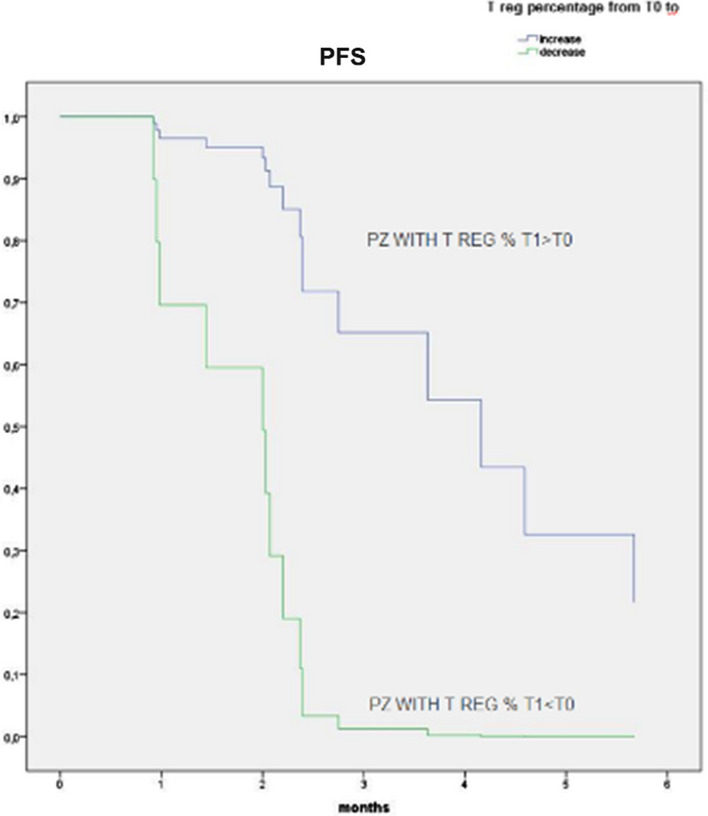
(Abstract 1) Cox analyses for PFS of patients with increased percentage of T_reg_ (blue line, n = 9) and patients with increased percentage of T_reg_ (green line, n = 9). This Figure 4 belongs to Abstract 1

**Conclusions:** Our results show that nivolumab could increase the levels of CCL-4 from T0 to T2. TNF-α and IFN-γ increased at TPD. Moreover, combination of IL-5, IL-6, CD3^+^CD8^+^LAG3^+^, CD3^+^CD8^+^PD1^+^LAG3^+^ and CD8^+^TEMRA basal levels might predict which patients could benefit from the treatment. Also, the increasing of T_reg_ percentages during treatment could be biomarker for poor prognosis.

**Acknowledgements**

This study is presented on behalf of NIVACTOR study group.

**References**

1. Argiris A, Karamouzis MV, Raben D, et al. Head and neck cancer. Lancet 2008; 371:1695–709.2
2. Chaturvedi AK, Engels EA, Anderson WF, et al. Incidence trends for human papilloma virus related and -unrelated oral squamous cell carcinomas in the United States. J Clin Oncol2008; 26:612–9.
3. Robert L. Ferris, George Blumenschein, Jerome Fayette et al. Nivolumab for Recurrent Squamous-Cell Carcinoma of the Head and Neck N Engl J Med 2016; 375:1856–1867.

## 2. Sequential therapy in stage IV melanoma – real-world SECOMBIT

### Teresa Amaral^1,2^*, Olivia Seber^1^, Stephanie Sanchez^1^, Ulrike Keim^1^, Ioannis Thomas^1^, Andreas Meiwes^1^, Andrea Forschner^1^, Thomas Eigentler^1^, Claus Garbe^1^

#### ^1^Centre for Dermatooncology, Department of Dermatology, Eberhard Karls University, Tuebingen, Germany; ^2^Portuguese Air Force Health Care Direction, Lisbon, Portugal

##### **Correspondence:** Teresa Amaral - Teresa.Amaral@med.uni-tuebingen.de

*Journal of Translational Medicine* 2020; **19(Supp 1):** 2.

**Background:** The approval of immune checkpoint inhibitors (ICI) and targeted therapies (TT) have changed the outcome of stage IV melanoma. Yet, data from clinical trials on the best therapy sequence are missing, particularly in patients (pts) with BRAFV600 mutated melanoma.

**Material and methods:** Here we analyzed pts diagnosed with stage IV melanoma between January 2015 and December 2018 in the Center for Dermato-Oncology of the Tuebingen University, and treated with ICI 1^st^ and 2^nd^ line (ICI-ICI); ICI 1^st^ line and TT 2^nd^ line (ICI-TT); TT 1^st^ line and ICI 2^nd^ line (TT-ICI) and TT 1^st^ and 2^nd^ line (TT-TT). Follow-up time was defined as the time between stage IV diagnosis and dead or last contact. We performed descriptive analysis of pts characteristics for the subgroups included and overall survival (OS) analysis.

**Results:** A total of 530 pts with stage IV melanoma were analyzed and 151 received two therapy lines. The median follow-up was 23 months (M) (95%CI: 20.5–25.5).

The majority of the pts received ICI-ICI (n = 62), 45 pts received TT-ICI, 34 pts received ICI-TT and only 10 pts received TT-TT. For pts in the subgroup ICI-ICI the 1^st^ line therapy was PD-1 monotherapy (n = 40); PD1 + CTLA4 (n = 10); and CTLA4 monotherapy (n = 12). In the sequence ICI-TT 1^st^ line treatment was as follows: PD-1 monotherapy (n = 21); PD1 + CTLA4 (n = 12); and CTLA4 monotherapy (n = 1).

When comparing the sequence (Person chi-square test) ICI-TT vs TT-ICI there were no statistically differences between the groups regarding sex, age, presence of elevated LDH or protein S100 at the time of diagnosis, number of organs involved and presence of brain or liver metastasis. However, a higher percentage of pts receiving TT-ICI compared to ICI-TT had elevated LDH (47% vs 29%); elevated protein S100 (60% vs 53%) and liver metastases (31% vs 26%) at the time of stage IV diagnosis.

The median OS for the whole collective was 22 M, 18 M for the sequences ICI-TT and TT-ICI; 28 M for the sequence ICI-ICI, and 19 M for the sequence TT-TT. There was no statistically significant difference in terms of OS between the 4 sequences (*p* = 0.084). When looking into the sequences ICI-TT and TT-ICI, the difference in OS was also not statistically significant (*p* = 0.261).

**Conclusions:** Second-line therapies seem to have a modest impact in OS when pts progress under 1^st^ line therapy. There was no significant difference in terms of OS between the four sequences.

## 3. Neoadjuvant nivolumab in early stage colon cancer induces promising tumor regression and T cell infiltration

### Antonio Avallone^1^*, A. De Stefano^1^, U. Pace^2^, A. Catteau^3^, E. Di Gennaro^4^, F. Tatangelo^5^, I. Boquet^3^, A. Kassamba^3^, A. Cassata^1^, S. Costantini^4^, S. De Franciscis^2^, F. Collina^5^, N. Zanaletti^1^, C. Vitagliano^4^, V. Granata^6^, D. Giannarelli^7^, S. Lastoria^8^, Paolo Antonio Ascierto^9^, Jerome Galon^10^, P. Delrio^2^, Alfredo Budillon^4^*

#### ^1^Experimental Clinical Abdominal Oncology, Istituto Nazionale Tumori Fondazione G. Pascale IRCCS, Napoli, Italy; ^2^Colorectal Surgery, Istituto Nazionale Tumori Fondazione G. Pascale IRCCS, Napoli, Italy; ^3^HalioDx, Luminy Biotech Enterprise, Marseille Cedex, France; ^4^Experimental Pharmacology Unit, Istituto Nazionale Tumori Fondazione G. Pascale IRCCS, Napoli, Italy; ^5^Phatology, Istituto Nazionale Tumori Fondazione G. Pascale IRCCS, Napoli, Italy; ^6^Radiology, Istituto Nazionale Tumori Fondazione G. Pascale IRCCS, Napoli, Italy; ^7^Unità di Biostatistica, IFO-Istituto Nazionale per lo Studio e la Cura dei Tumori Regina Elena – Roma, Italy; ^8^Nuclear Medicine, Istituto Nazionale Tumori Fondazione G. Pascale IRCCS, Napoli, Italy; ^9^Cancer Immunotherapy and Development Therapeutics, Istituto Nazionale Tumori Fondazione G. Pascale IRCCS, Napoli, Italy; ^10^Cordeliers Research Center, INSERM team 15, Laboratory of Integrative Cancer Immunology, Paris, France

##### **Correspondence:** Antonio Avallone - a.avallone@istitutotumori.na.it; Alfredo Budillon - a.budillon@istitutotumori.na.it

*Journal of Translational Medicine* 2020; **19(Supp 1):** 3.

**Background:** Neoadjuvant treatment is well established in early stage solid tumors but few trials have been conducted in colon cancer. In mismatch repair deficient (dMMR) colon cancer patients programmed death 1 (PD-1) blockade is highly effective in metastatic setting and plus cytotoxic T lymphocyte antigen-4 (CTLA-4) blockade induced high pathological response rate in early stages.

**Material and Methods:** NICOLE is the first neoadjuvant study to test anti PD-1 nivolumab in unselected MMR early stage resectable colon cancer (T3-T4) patients (NCT04123925). Primary endpoints were safety, feasibility, pathological tumor response, molecular and immunophenotypic studies. 22 patients (19 pMMR and/or MSS, 3 dMMR and/or MSI) received nivolumab at 240 mg flat dosage on day 1 + 15, and underwent surgery within 3 weeks thereafter without delay or complications.

**Results:** Major pathological responses (≤ 20% viable tumor cells) were observed in 3 pMMR/MSS, including 1 complete response, and in 1 pMMR/MSI tumors. In additional 4 confirmed MSS tumors we observed ≥ 30% tumor regression. A clear downstaging was observed in more than 70% of pts. Double blind pathological evaluation is ongoing. At surgery tumors from NICOLE cohort showed significantly higher level of CD8 T-cells infiltration (p =  < 0.035) and significant higher Immunoscore (densities of CD3 + and CD8 + T-cells in tumor and invasive margin regions) (p = 0.028), compared to a control cohort of 22 colon cancer patients undergoing surgery without treatment. Moreover, a significant increase in tumor CD8 T-cells infiltration was seen at surgery compared to pre-treatment biopsy (p =  < 0.0015) in the NICOLE cohort. On the contrary no significant differences were observed between baseline and preoperative radiological and metabolic assessment. Analysis of blood biomarkers (cytokines, metabolomics, immune cell subpopulations, tumor immune gene signature and microbioma profiling) are ongoing.

**Conclusions:** Overall, neoadjuvant nivolumab in early stage colon cancer patients is feasible, do not compromise surgery and leads to an increase of tumor T-lymphocytes infiltration. The promising tumor regression observed in pMMR/MSS patients, suggest a potential of preoperative combination treatment with immunotherapy in this setting.

## 4. Safety and Efficacy of TRIplet combination of Nivolumab (N) with Dabrafenib (D) and Trametinib (T) [TRIDeNT] in Patients (pts) with BRAF-mutated Metastatic Melanoma (MM): a Single Center Phase II Study

### Elizabeth M. Burton^1^*, Rodabe N. Amaria^1^, Isabella Claudia Glitza^1^, Adi Diab^1^, Denài R. Milton^2^, Sapna Pradyuman Patel^1^, Jennifer McQuade^1^, Virginia Honaker^1^, Courtney Brown^1^, Michael K. K. Wong^1^, Patrick Hwu^3^, Jennifer Wargo^4^, Michael A. Davies^1^, Hussein Tawbi^1^.

#### ^1^The University of Texas MD Anderson Cancer Center, Department of Melanoma Medical Oncology, Houston, TX, US; ^2^The University of Texas MD Anderson Cancer Center, Department of Biostatistics, Houston, TX, US; ^3^Moffitt Cancer Center, Tampa, FL, US; ^4^The University of Texas MD Anderson Cancer Center, Department of Surgical Oncology, Houston, TX, US.

##### **Correspondence:** Elizabeth M. Burton - emburton@mdanderson.org

*Journal of Translational Medicine* 2020; **19(Supp 1):** 4.

**Background:** Although targeted therapies (TT) and immunotherapies (IMT) have improved survival for pts with BRAF V600 mutated stage IV MM, many pts progress and will ultimately die from their disease. Preclinical data has shown that BRAF inhibition (BRAFi) in BRAF-mutated tumors is associated with increased T cell infiltration, supporting the rationale for a clinical combinatorial approach with IMT. Although trials evaluating triplet combinations in the IMT naïve setting have reported mixed results, with one triplet gaining FDA approval, there are no approved therapies for pts *after* IMT failure. Notably, pts with untreated brain metastases (BM) are also excluded from such trials. We hypothesized that N in combination with DT is safe and will demonstrate clinical activity in BRAF-mutated pts naïve or refractory to PD1 therapy and in pts with BM.

**Methods:** We report a single arm phase II study (NCT02910700) of NDT in pts with BRAF-mutated, unresectable stage III or stage IV MM. Prior IMT was allowed, but pts who received prior BRAF/MEKi were ineligible. Pts with untreated BM and asymptomatic or mildly symptomatic/requiring stable or decreasing steroids (up to PO dexamethasone of 8 mg or equivalent) were also allowed. Pts received 3 mg/kg Q2wks of N (later amended to 480 mg q4wks), 150 mg BID of D and 2 mg QD of T, all starting on Day 1. The primary objective is to determine safety and efficacy (ORR by RECIST 1.1) of the NDT combination. We performed continuous monitoring for safety and futility using Bayesian stopping rules. Longitudinal tissue and blood samples were collected to assess for future correlative analyses.

**Results:** Following a 6 pt safety run-in with no observed DLTs, 27 pts received NDT—17 pts were PD1 refractory, 10 were PD-1 naïve. 10 of these 27 pts had a history or presence of BM. Median follow up was 18.4mos (3.2–45.9). ORR for 26 evaluable pts was 92%, including 3 pts who achieved a CR. 16 PD1 refractory pts were evaluable for response; 2 achieved CR and 12 PR (ORR 88%). Each of the 10 evaluable PD-1 naïve pts achieved a response. 4 of the 7 evaluable pts with a BM achieved an intracranial response (57%), including 2 CRs. The median PFS for all pts was 8.5 months – (8.5mos in PD1 naïve pts and 8.2mos in PD1 refractory pts), the median OS was not reached. Median PFS for pts without BM was 8.5mos and 8mos for those with BM. 78% of pts experienced treatment related grade 3/4 AEs and only 6 pts discontinued due to toxicities.

**Conclusion:** NDT at full doses of all 3 agents has a toxicity profile consistent with previously reported triplet combinations and shows promising clinical activity in pts with IMT refractory disease and with BM. There were no significant differences in outcomes between pts with and without BM. Further translational investigation to better delineate mechanisms of response are ongoing.

## 5. Progression-free survival and biomarker correlates of response with BEMPEG plus NIVO in previously untreated patients with metastatic melanoma: results from the PIVOT-02 study

### Adi Diab^1^*, Scott S. Tykodi^2^, Gregory A. Daniels^3^, Michele Maio^4^, Brendan D. Curti^5^, Karl D. Lewis^6^; Sekwon Jang^7^, Ewa Kalinka^8^, Igor Puzanov^9^, Alexander I. Spira^10^, Daniel C. Cho^11^, Shanhong Guan^12^, Erika Puente^12^, Ute Hoch^12^, Sue L. Currie^12^, Tuan Nguyen^12^, Wei Lin^12^, Mary A. Tagliaferri^12^, Jonathan Zalevsky^12^, Mario Sznol^13^, Michael E. Hurwitz^13^

#### ^1^The University of Texas MD Anderson Cancer Center, Houston, TX, USA; ^2^Seattle Cancer Care Alliance, Seattle, WA, USA; ^3^University of California, La Jolla, CA, USA; ^4^Azienda Ospedaliera Universitaria Senese, Siena, Italy; ^5^Providence Cancer Center and Earle A. Chiles Research Institute, Portland, OR, USA; ^6^University of Colorado, Aurora, CO, USA; ^7^Inova Schar Cancer Institute, Fairfax, VA, USA; ^8^Polish Mother’s Memorial Hospital – Research Institute, Lodz, Poland; ^9^Roswell Park Comprehensive Cancer Center, Buffalo, NY, USA; ^10^Virginia Cancer Specialists, Fairfax, VA, USA; ^11^NYU Medical Oncology Associates, New York, NY, USA; ^12^Nektar Therapeutics, San Francisco, CA, USA; ^13^Yale School of Medicine, New Haven, CT, USA

##### **Correspondence:** Abi Diab - adiab@mdanderson.org

*Journal of Translational Medicine* 2020; **19(Supp 1):** 5.

**Background:** An unmet need exists for novel therapies that produce deep and durable responses in more patients with metastatic melanoma (metMEL). Encouraging clinical activity was observed with the CD122-preferential IL-2 pathway agonist bempegaldesleukin (BEMPEG) plus nivolumab (NIVO) in first-line metMEL in the phase 1/2 PIVOT-02 trial (NCT02983045)[1], leading to FDA Breakthrough Therapy Designation. We present updated clinical results from PIVOT-02 (NCT02983045) in first-line metMEL, and biomarkers of response.

**Methods:** 41 patients with previously untreated stage IV melanoma (known PD-L1 status by immunohistochemistry; 28–8 PharmDx) received ≥ 1 dose of BEMPEG (0.006 mg/kg) plus NIVO (360 mg) q3wks; 38 patients were efficacy-evaluable (≥ 1 post-baseline tumor scan). Primary endpoints were safety and objective response rate (ORR; RECIST v1.1; BICR); other endpoints included PFS, OS and biomarkers. Polyfunctional strength index (PSI) of circulating lymphocytes (determined using single-cell cytokine analysis [Isoplexis]) and eosinophil count (determined from hematology analysis) at baseline and Cycle 1-Day 8 were analyzed using the median cut-off for correlations with ORR and PFS. Biomarkers, including CD8^+^ tumor infiltrating lymphocytes (TIL) and interferon-gamma (IFNg) gene expression profile (GEP), were measured in baseline tumor biopsies and analyzed for correlation with ORR and PFS.

**Results:** At median follow-up of 29.0 months (1Sept2020), ORR by BICR was 53% (20/38 patients). Complete response occurred in 13/38 patients (34%): 23% PD-L1-negative (< 1% tumor cell expression); 41% PD-L1-positive (≥ 1% tumor cell expression). Further deepening of response was observed, with 18/38 patients (47%) achieving 100% reduction in target lesions and a 79% median reduction from baseline in tumor size (previously 62% [1]). Median time to response and time to complete response was 2.0 and 7.9 months, respectively. Median PFS was 30.9 months (95% CI: 5.3; NE; ITT). Median OS was not reached; 2-year OS rate was 77% (95% CI: 60–87; ITT). Safety was consistent with previous reports[1].

IFNg GEP and CD8^+^ TIL in baseline tumor biopsies were significantly associated with ORR and PFS. Analysis of Cycle 1-Day 8 blood samples demonstrated significant increases in CD4^+^PSI, CD8^+^PSI, and eosinophils from baseline. Increased CD8^+^PSI was significantly associated with higher ORR and PFS; increased eosinophils were significantly associated with higher ORR.

**Conclusions:** BEMPEG plus NIVO was well tolerated in first-line metMEL. Responses were deep and durable, with a complete response rate of 34% and a median PFS of 30.9 months. Non-invasive, on-treatment biomarkers predicted response, well before radiologic evidence. A phase 3 trial evaluating BEMPEG plus NIVO in first-line metMEL is enrolling(NCT03635983).

**Acknowledgements**

This study was supported by Nektar Therapeutics.

**Trial registration**

NCT02983045

**Reference**

1. Diab A, Puzanov I, Maio M, et al. Clinical activity of BEMPEG plus NIVO in previously untreated patients with metastatic melanoma: updated results from the phase 1/2 PIVOT-02 study. Oral presentation at SITC; November 6–10, 2019; National Harbor, MD, USA. Abstract #O35.

## 6. Pretreatment neutrophil–lymphocyte ratio (NLR) predicted overall survival (OS) for metastatic melanoma, non-small cell lung cancer (NSCLC), renal cell carcinoma (RCC) patients who received monotherapy with immune checkpoint inhibitor as ipilimumab, nivolumab and pembrolizumab

### Pietro Di Marino^1^*, Francesca Chiara Primavera^1^, Maria Teresa Martino^1^, Consiglia Carella^1^, Antonino Grassadonia^2^, Nicola Tinari^2^, Clara Natoli^2^, Michele De Tursi^2^.

#### ^1^Clinical Oncology Unit, S.S. Annunziata Hospital, 66,100 Chieti, Italy^1^ Clinical Oncology Unit, S.S. Annunziata Hospital, 66,100 Chieti, Italy; ^2^Department of Medical, Oral and Biotechnological Sciences and Center for Advance Studies and Technology (CAST), G. D'Annunzio University, 66,100 Chieti, Italy

##### **Correspondence:** Pietro Di Marino - pietrodimarino@gmail.com

*Journal of Translational Medicine* 2020; **19(Supp 1):** 6.

**Background:** Immunotherapy has become the standard of care for an increasing number of malignancies. Cancer related inflammatory process has been shown to have an important role in patient prognosis. High platelet-to-lymphocyte ratio (PLR) and neutrophil-to-lymphocyte ratio (NLR) are markers of host inflammation and are associated with worse overall survival (OS) in several cancers. The relationship between immunotherapy, PLR and NLR is poorly understood. The aim of this study is to investigate the association of NLR and OS and the association of PLR and OS in patients receiving immune checkpoint blockade for metastatic melanoma, non-small cell lung cancer (NSCLC) and renal cell carcinoma (RCC).

**Materials and methods:** We conducted a retrospective study of metastatic melanoma, NSCLC, RCC patients treated with ipilimumab, nivolumab or pembrolizumab. NLR was defined as absolute neutrophil count divided by absolute lymphocyte count in peripheral blood, PLR was defined as absolute platelet count divided by absolute lymphocyte count in peripheral blood. We examined NLR and PLR at baseline, when the first administration of immunotherapy was performed.

**Results:** 113 patients (89♂ 24 ♀) received immunotherapy from October 2013 to April 2020 in our hospital. The data cut-off analysis was August 2020. Median age was 64 years (27–85). 50 (44,2%) patients received ipilimumab or nivolumab or pembrolizumab for melanoma, 50 (44,2%) patients received nivolumab or pembrolizumab for NSCLC, 13 (11,6%) patients received nivolumab for RCC. Of these patients, receiver operating characteristic (ROC) curves analysis were used to confirm the cut-off value, and patients were stratified into NLR < 5 (65♂ 20♀) and NLR** ≥ **5 (24♂4 ♀) groups and stratified into PLR < 200 (59♂ 12♀) and PLR ≥ 200 (30♂12 ♀) groups. The median OS was 14.8 months (1.0 to 62.5 months). When NLR < 5, patients had significantly longer OS: 17,4 months vs 6,8 months (p < 0,0001). No difference statistically significant was found between PLR < 200 and PLR ≥ 200 group for OS (p = 0,182).

**Conclusions**: NLR could be a cheap and easily available prognostic marker in metastatic melanoma, NSCLC, RCC patients receiving monotherapy of immune checkpoint inhibitor as ipilimumab, nivolumab or pembrolizumab.

## 7. Clinical biomarkers of response to anti-PD-1 first-line treatment in an Italian patient cohort

### Francesca Romana Di Pietro^1^*, Sofia Verkhovskaia^1^, Simona Mastroeni^2^, Maria Luigia Carbone^3^, Damiano Abeni^2^, Zorika Christiana Di Rocco^1^, Nicola Samà^1^, Albina Rita Zappalà^1^, Paolo Marchetti^4,5^, Federica De Galitiis^1^, Cristina Maria Failla^3^, Cristina Fortes^2^

#### ^1^Department of Oncology and Dermatological Oncology, IDI-IRCCS, Rome, Italy; ^2^Epidemiology Unit, IDI-IRCCS, Rome, Italy; ^3^Experimental Immunology Laboratory, IDI-IRCCS, Rome, Italy; ^4^Department of Clinical and Molecular Medicine, Oncology Unit, Sant'Andrea Hospital, Sapienza University, Rome, Italy; ^5^Medical Oncology Unit B, Policlinico Umberto I, Sapienza University, Rome, Italy

##### **Correspondence:** Francesca Romana Di Pietro - francescadipietro27@gmail.com

*Journal of Translational Medicine* 2020; **19(Supp 1):** 7.

**Background:** Melanoma is one of the most immunogenic tumors and immunotherapy treatment with checkpoint inhibitors (ICI), such as anti-programmed cell death protein (PD)-1 antibodies, has significantly improved the prognosis of metastatic melanoma. However, only half of the patients responds to this therapy and has a favorable outcome[1]. Understanding the factors responsible for treatment failure and early identification of responders are both important to select the best therapeutic strategy for each patient. Thus, the aim of our study was to investigate clinical biomarkers of response to treatment with anti-PD-1 antibodies.

**Materials and methods:** We identified all patients with inoperable stage III or stage IV melanoma (N = 147), subjected to first-line treatment with anti-PD-1 at IDI-IRCCS in the last 10 years. We studied the associations between patients’ different clinical features and progression free survival (PFS), using the COX proportional hazard models.

**Results:** In the multivariate analysis, an increased risk of disease progression was observed among patients with stage M1d,who developed brain metastases(with HR 2.92; 95% CI 1.38–618), compared to patients with stage M1a-M1b with skin, subcutaneous, soft tissue, lymph node and/or lung lesions. Moreover, the risk of progression was greater in patients with the Eastern Cooperative Oncology Group Performance Status (ECOG PS) 1 (HR:2.67; 95% CI: 1.30–5.52), and in patients with ECOG PS ≥ 2 (HR:2.21; 95%CI:1.06–4.63) with respect to PS ECOG 0. High levels of lactate dehydrogenase (LDH) at baseline was associated with a major risk of disease progression (HR 2.09; 95% CI: 1.16–3.75) (Table 1). In a sub-group analysis (48 patients), we evaluated neutrophil count, the neutrophil/lymphocyte ratio (NLR) and the derived (d)NLR before the beginning of anti-PD-1 therapy and we observed that these values ​​were higher among disease progression patients, with respect to those who responded to therapy.

**Table 1:** (Abstract 7) Clinical biomarkers of response to anti-PD-1 and progression free survival. This Table 1 belongs to Abstract 7

	Univariate		Multivariate*	
	Types of Melanoma (cutaneous, mucosal, uveal)		Types of Melanoma (cutaneous, mucosal, uveal)	
	Crude HR (95%CI)	*P*	Adjusted HR (95%CI)	*P*
ECOG performance status				
0	1		1	
1	2.13 (1.09–4.16)	0.026	2.67 (1.30–5.52)	0.008
≥ 2	3.08 (1.56–6.05)	0.001	2.21 (1.06–4.63)	0.035
Stages of metastasis				
1a-1b	1		1	
1c	1.88 (1.04–3.38)	0.036	1.80 (0.95–3.41)	0.074
1d	3.18 (1.66–6.08)	< 0.0001	2.92 (1.38–6.18)	0.005
LDH serum				
Normal	1		1	
High	2.32 (1.34–4.01)	0.003	2.09 (1.16–3.75)	0.014

HR, Hazard Ratio; CI, Confidence Interval

* the estimates are adjusted for gender, age, ECOG PS, stage of metastasis and LDH levels

**Conclusions:** In our study population, the main independent predictors of diseases progression among patients treated with anti PD-1 first-line were as following: ECOG PS, staging and LDH. Further studies are warranted to confirm our findings.

**Ethics Approval:**

For this study, approval was obtained from the Ethics Committee of the IDI-IRCCS (Opinion Register No. 510/5 and subsequent amendments).

**Reference**

1. Passarelli A, Mannavola F, Stucci LS, Tucci M, Silvestris F. Immune system and melanoma biology: a balance between immunosurveillance and immune escape. Oncotarget. 2017; 62:106,132–10,614

## 8. Reverse transcriptase inhibition potentiates target therapy in BRAF-mutant melanomas: an in vitro study

### Luigi Fattore^1,2^*, Gianluca Sbardella^3^, Gerardo Botti^2^, Rita Mancini^4^, Paolo A. Ascierto^2^ and Gennaro Ciliberto^5^

#### ^1^SAFU Laboratory, Department of Research, Advanced Diagnostics and Technological Innovation, Translational Research Area, IRCCS Regina Elena National Cancer Institute, Rome, Italy; ^2^Istituto Nazionale Tumori IRCCS, “Fondazione G. Pascale”, Naples, Italy; ^3^Department of Pharmacy, Epigenetic Med Chem Lab, University of Salerno, Fisciano, SA, Italy; ^4^Department of Molecular and Clinical Medicine, University of Roma “Sapienza”, Rome, Italy; ^5^IRCCS, Istituto Nazionale Tumori “Regina Elena”, Scientific Directorate, Rome, Italy

##### **Correspondence:** Luigi Fattore - luigifattore1985@gmail.com

*Journal of Translational Medicine* 2020; **19(Supp 1):** 8.

**Background:** BRAF + MEK inhibitors have become the standard of care for melanoma patients (approximately 50%) harboring BRAF-V600 mutations. However, drug resistance often impairs the efficacy of this combinatorial approach. Hence, the need to identify additional therapeutic approaches capable to control the development of drug resistance and to avoid disease relapse. Towards this goal, our group has been largely involved in the last years in the study of non-mutational mechanisms involved in the acquisition of drug resistance. For example, we reported that monoclonal antibodies targeting ErbB3 receptor are able to delay the emergence of resistance to target therapy in vitro and in vivo. More recently, we have also demonstrated that microRNAs are key players of resistance to target therapy in melanoma and that their targeting is able to restore drug sensitivity. Here, we have started to investigate whether reverse transcriptase inhibitors (RTIs) frequently used in the treatment of AIDS can act in combination with target therapy to fight the development of drug resistance.

**Material and methods:** Human melanoma cell lines, namely M14 and A375 harboring BRAF-V600E mutation have been treated with different concentration of either BRAFi and/or MEKi in the presence or not of the non-nucleoside RTI, i.e. SPV122. MTT and colony formation assays have been used to determine cell proliferation. Annexin V assay for apoptosis, cell cycle and mitochondrial membrane depolarization have been tested through FACS analyses. DNA double-strand breaks have been determined through Western Blot and Immunofluorescence analyses.

**Results:** Our present work has reported for the first time the capability of RTIs to mitigate drug resistance to target therapy in BRAF-mutant melanomas in vitro. We show that the novel non-nucleoside RTI, SPV122 synergizes with BRAF and MEK inhibitors to: 1) impair BRAF-mutant melanoma cell growth; 2) induce apoptosis; 3) block cell cycle progression and 4) delay the emergence of resistance in vitro. Mechanistically, we also show that this combination provokes DNA double-strand breaks, mitochondrial membrane depolarization and increased ROS levels.

**Conclusions:** Our in vitro results pave the way for the combinatorial use of RTi + BRAFi + MEKi as a novel therapeutic option for BRAF-mutant melanoma patients and warrant further investigation in in vivo models.

## 9. Large-scale proteomic analysis of soluble compartment for biomarkers discovery in pediatric acute lymphoblastic leukemia

### Giusy Gentilcore^1^*, Sara Deola^1^, Sheanna Maria Herrera^1^, Che-Ann Lachica^1^, Tayseer Abbas Yousif^2^, Areeg Ahmed^2^, Naima Al Mulla^2^, Ayman Saleh^2^, Tommaso Mina^3^, Patrizia Comoli^3^, Jean-Charles Grivel^4^, Chiara Cugno^1*^

#### ^1^Advanced Cell Therapy Core, Sidra Medicine, Doha, Qatar; ^2^Pediatric Hematology and Oncology Department, Sidra Medicine, Doha, Qatar; ^3^Pediatric Oncology and Hematology Department, IRCCS Fondazione Policlinico San Matteo, Pavia, Italy; ^4^Deep Phenotyping Core, Sidra Medicine, Doha, Qatar

##### **Correspondence:** Giusy Gentilcore - ggentilcore@sidra.org

*Journal of Translational Medicine* 2020; **19(Supp 1):** 9.

**Background:** Acute Lymphoblastic Leukemia (ALL) is the most common malignancy in children and represents 75–80% of leukemia cases. Despite its recognized role in the initiation of the disease, the bone marrow (BM) microenvironment is not well understood especially in its non-cellular component.

High-throughput protein detection represent an opportunity to unveil new biomarkers for leukemia diagnosis, prognosis, monitoring, and to potentially identify biological targets.

**Materials and methods:** In this study, we evaluated the soluble compartment of BM and peripheral blood (PB) in 16 ALL pediatric patients by the means of the SomaScan assay, a highly multiplexed, affinity proteomics platform able to measure 1305 proteins in 65 µl of sample using modified protein-binding single-stranded DNA aptamers called SOMAmers, revealed on a high dimension oligo-probe array. The raw hybridization data were normalized using hybridization controls and were log2-transformed. Transformed HybNormalized BM and PB plasma data were compared using GraphPad PRISM 8. Multiple comparisons were set with an FDR threshold of 0.01%.

**Results:** The comparative analysis between plasma samples from BM and PB showed that 228 proteins are differentially expressed in BM and PB plasma irrespective of the nature of disease (Table 1). A more detailed mix-effect analysis of protein expression in the different patient categories and compartment defined in Table 1 revealed that out of these 228 proteins, 92 showed a significant differential expression when performing a mixed-effect analysis between the different groups. Among the top 20 proteins differentially expressed between BM and PB, 18 show differential expression between patient groups. The patient group effect is dominated by the differences between BM and PB plasma in ALL Common patients, which account for 17 of the observed differences in protein levels. In spite of the limited sample size, the analysis revealed a difference in NOTC2 level between ALL Common PB and ALL Pro-B PB.

**Conclusion:** In our study we applied a quantitative large-scale proteomic analysis on BM and PB plasma in children suffering from ALL, identifying differential protein expression between the two compartments.

In the last few years, SomaScan has emerged as a very attractive method due to its high throughput capacity, faster discovery mode compared to other proteomic platforms and small sample size required, which make it ideal for the identification of novel biomarkers. In fact, differences in protein expression were revealed in these limiting samples sizes. We are taking this analysis further by analyzing more patients in different groups and including normal donor samples.

**Table 1:** (Abstract 9) This Table 1 belongs to Abstract 9

Pts Code	Age (yrs)	Phenotype	Cytogenetics	Bone Marrow	Peripheral Blood
ALL#02P	1.5	B-cell ALL common	Normal	Yes	
ALL#03P	1.5	B-cell ALL common	Complex karyotype	Yes	
ALL#05P	15	B-cell ALL common	Normal	Yes	
ALL#09P	-	T-cell ALL	Normal	Yes	
ALL#10P	8	B-cell ALL common	Normal	Yes	Yes
ALL#11P	12	B-cell ALL common	Normal	Yes	Yes
ALL#12P	5	Pro-B ALL	Normal		Yes
ALL#13P	11	B-cell ALL common	t(1;19)		Yes
ALL#14P	12	Pro-B ALL	Normal		Yes
ALL#15P	7	T-cell ALL	Normal		Yes
ALL#16P	16	B-cell ALL common	Normal	Yes	Yes
ALL#17P	15	T-cell ALL	Normal	Yes	
ALL#18P	1.5	B-cell ALL Common	Normal	Yes	
ALL#19P	3	B-cell ALL Common	t(12;21)		Yes
ALL#20P	5	B-cell ALL Common	t(12;21)		Yes
ALL#21P	14	ALL B Mature	t(8;14)	Yes	

## 10. Metastatic cutaneous melanoma epidemiological registry in Turkey: A preliminary evaluation of diagnosis and treatment approaches

### Burçak Ş. Karaca*^1^, Ahmet Sezer^2^, Sezgin S. Göksu^3^, İrfan Çiçin^4^, Dilek Erdem^5^, Erdem Cubukcu^6^, Faysal Dane^7^, Ilhan Hacıbekiroğlu^8^, Berna Öksüzoğlu^9^, Ebru Alnıgeniş^10^, Esat Ulay^10^, İsmail Çelik^11^

#### ^1^Ege University Hospital, Department of Internal Medicine, Division of Medical Oncology, Izmir, Turkey; ^2^Başkent University Adana Dr. Turgut Noyan Practice and Research Centre, Department of Medical Oncology, Adana, Turkey; ^3^Akdeniz University Faculty of Medicine, Department of Internal Medicine, Division of Medical Oncology, Antalya, Turkey; ^4^Trakya University Faculty of Medicine, Department of Internal Medicine, Division of Medical Oncology, Edirne, Turkey; ^5^Bahçeşehir University Faculty of Medicine, Department of Internal Medicine, Division of Medical Oncology, Istanbul, Turkey; ^6^Uludağ University Faculty of Medicine, Department of Internal Medicine, Division of Medical Oncology, Bursa, Turkey; ^7^Marmara University Istanbul Pendik Training and Research Hospital, Department of Internal Medicine, Division of Medical Oncology, Istanbul, Turkey; ^8^Sakarya University Training and Research Hospital, Department of Internal Medicine, Division of Medical Oncology, Sakarya, Turkey; ^9^Dr. Abdurrahman Yurtaslan Ankara Oncology Training and Research Hospital, Medical Oncology Clinic, Ankara, Turkey; ^10^Novartıs Oncology, Istanbul, Turkey; ^11^Hacettepe University Faculty of Medicine, Department of Internal Medicine, Division of Medical Oncology, Ankara, Turkey

##### **Correspondence:** Burçak Ş. Karaca - karacaburcak@hotmail.com

*Journal of Translational Medicine* 2020; **19(Supp 1):** 10.

**Background:** Turkey has some limits in reaching some new treatment options. Our aim was to determine the current diagnostic and treatment approaches of metastatic cutaneous melanoma (MCM) in ten main oncology clinics in Turkey. To the best of our knowledge, this is the first epidemiological registry study specific for MCM status in Turkey.

**Materials and methods:** This was a cross-sectional, retrospective epidemiological registry study. Data was collected from patients' file records. Patients with MCM diagnosis between December 2017 and June 2019 were eligible.

**Results:** Totally 140 patients were eligible for the registry; 62.9% of them were male. Median age at the diagnosis was 56 years. Primary tumour localization was predominantly lower extremity (32.4%). The most common histological subtype was nodular melanoma (57.8%). Of the patients, 90.3% were classified with the Eastern Cooperative Oncology Group as 0 or performance status of 1. Primary tumour was found in 75% of the patients. From all cases, quarter of them had progressed from being stage III disease and 14% of them from being stage II at the time of diagnosis. The median Breslow thickness was 5 mm and ulceration was present in 37% of the patients. Sentinel lymph node dissection was not performed in about 80% of all cases. Of the patients, 31.9% had M1c disease 14.3% had brain metastasis at the time of MCM diagnosis. BRAF mutation status was mainly analysed by real-time polymerase chain reaction and was positive in 39.3% of the patients. Radiotherapy was applied mostly for palliative reasons (75%) and mostly for brain metastases. The most common first line treatment was temozolomide in 67% of all patients and this was followed by ipilimumab (33%) as a second line treatment. Nivolumab was only applied in 9.6% of the patients in the first line treatment and in 24.7% of the patients in the second line of treatment. Among BRAF inhibitors, dabrafenib and trametinib combination was the most preferred one; nearly 25% of the patients received this combination as a first line treatment.

**Conclusions:** Chemotherapy is preferred in the front line for BRAF wild type patients, which leads to worsened outcomes. Dabrafenib and trametinib combination is preferred for most of the BRAF mutant patients, but some patients could not receive any BRAF inhibitors. This study highlighted that there is still unmet need for early diagnosis of melanoma and the importance of access to effective treatment options for all melanoma patients.

## 11. Circulating levels of miR-204-5p predict response to BRAF and MEK inhibitors in metastatic melanoma patients

### Ciro F. Ruggiero^1^*, Irene Terrenato^2^ Luigi Fattore^2^, Valentina Salvati^2^, Francesca Sperati ^3^ Domenico Liguoro^4^, Vittorio Castaldo^4^, Gabriele Madonna^5^, Marialena Capone^5^,Antonio Grimaldi^5^, Diana Giannarelli^2^, Paolo A. Ascierto^5^, Rita Mancini^4^ and Gennaro Ciliberto^2^

#### ^1^Department of Experimental and Clinical Medicine, University “Magna Graecia” of Catanzaro, Catanzaro, Italy; ^2^IRCCS, Regina Elena National Cancer Institute, Rome, Italy; ^3^San Gallicano Dermatological Institute, IRCSS, Rome Italy; ^4^University of Roma “Sapienza”, Department of Clinical and Molecular Medicine Rome, Italy; ^5^Istituto Nazionale Tumori IRCCS, “Fondazione G. Pascale”, Naples, Italy

##### **Correspondence:** Ciro F. Ruggiero - cirofrancescoruggier@libero.it

*Journal of Translational Medicine* 2020; **19(Supp 1):** 11.

**Background:** Metastatic melanomas harboring BRAF-V600 mutations are currently treated with combinations of BRAF and MEK inhibitors (MAPKi) increasing the objective responses, disease free survival and overall survival over monotherapy with BRAF inhibitors. Unfortunately, several patients suffer from ab initio or acquired resistance to these agents. Several efforts have been directed in recent years to understand mechanisms of resistance to MAPK inhibitors. These studies have shown a prominent involvement of non-mutational adaptive events, among which also deregulation of miRNAs expression. In this regard our laboratory has identified several miRNAs which undergo either up- or down-regulation during the development of drug resistance [1]. In this study we have started to assess whether circulating levels of one or more of these miRNAs can act as an early predictor of response to therapy.

**Materials and methods:** Circulating miRNAs were extracted from the serum of 51 BRAF-mutated melanoma patients before the beginning of therapy through miRNeasy Mini Kit (Qiagen). Real-time PCR for miR-204-5p, miR-199b-5p miR-579-3p, miR-9-5p miR-4443 and miR-4488 was assayed by TaqMan Gene Expression. Data of circulating miRNAs were normalized using Global mean normalization and NormFinder model [2]. For analysis purposes, ∆Ct miRNA values were dichotomized on the basis of the cut-off established using the receiver operating characteristics (ROC) curve considering OS specific condition (alive/dead within 12 months from MAPKi therapy) as the state variable. Overall Survival (OS) and Progression Free Survival (PFS) analyses were carried out by the Kaplan–Meier product-limit method. The Log Rank test was used to prove if any statistically significant difference between subgroups exists (p-value < 0.05).

**Results:** This retrospective study involved patients with a median age of 45 years. Among them, 27 (53%) were females. All patients were treated with MAPKi therapy in first line. Only miR-204-5p emerged to have a role in predicting both OS and PFS. Concerning OS, patients with a ∆Ct value under the ROC cut-off show a shorter median time to death in comparison to patients with a ∆Ct value over the ROC cut-off (10 months 95% confidence interval (95%CI): (3.9–16.1) vs 34 months 95%CI: (25.7–42.3); p-value = 0.013). Concerning PFS analysis, patients with a ∆Ct value under the ROC cut-off have a shorter median time to progression in comparison to patients with a ∆Ct value over the ROC cut-off (5 months 95%CI: (4.1–5.9) vs 18 months 95%CI: (7.9–28.1); p-value = 0.006).

**Conclusions:** On the basis of these results, miR-204-5p can be a promising predictive biomarker able to discriminate advanced melanoma patients who may benefit of MAPKi treatments. These data warrants of further validation in an extended cohort of patients as well as in prospective following studies.

**Bibliography**

1. Fattore L, et al. Cell Death Differ. 2019. https://doi.org/10.1038/s41418-018-0205-5.
2. Fogli S, et al. Tumour Biol. 2017; https://doi.org/10.1177/1010428317701646.

## 12. Characterization of DNA cargo of exosomes derived from irradiated breast cancer cells

### Sheila Spada^1^*, Paul Zumbo^2^, Doron Betel^2^, Nils-Petter Rudqvist^1^, Tuo Zhang^3^, Sandra Demaria^1^

#### ^1^Department of Radiation Oncology, Weill Cornell Medicine, New York, NY, USA; ^2^Applied Bioinformatics Core, Weill Cornell Medicine, New York, NY, USA; ^3^Genomics Resources Core Facility, Weill Cornell Medicine, New York, NY, USA

##### **Correspondence:** Sheila Spada - shs2066@med.cornell.edu

*Journal of Translational Medicine* 2020; **19(Supp 1):** 12.

**Background:** Immunogenic dose (8GyX3) of radiation determines an increase of cytosolic DNA that is sensed by cGAS leading to downstream activation of interferon type I (IFN-I) signaling in breast cancer cells. [1–3]. Our previous report demonstrated that irradiated TSA breast cancer cells release tumor-derived exosomes (RT-TEX) containing a DNA cargo that stimulates IFN-I production in recipient dendritic cells (DCs) via the cGAS/STING pathway, whereas exosomes secreted by untreated cells (UT-TEX) contain DNA that is unable to stimulate IFN-I [4]. Furthermore, RT-TEX, but not UT-TEX, when used to vaccinate mice stimulated anti-tumor immune response preventing tumor growth [4]. Here, we hypothesized that the differential capability of RT-TEX and UT-TEX to activate IFN-I in recipient DCs depends on qualitative differences in the DNA cargo of RT-TEX versus UT-TEX.

**Materials and methods:** Agilent Bioanalyzer system was used to measure the length of DNA purified from TEX and from the cytosolic fraction of TSA cells. Whole-genome sequencing (WGS) and whole-genome bisulfite sequencing were performed to analyze the DNA cargo of TEX. 5-methyl cytosine DNA Elisa kit was used to quantify the percentage of methylation of total DNA in TSA cells.

**Results:** Enrichment of DNA fragments with size between 60 and 250 bp was found in RT-TEX compared to UT-TEX, as well as in the cytosolic fraction of irradiated compared to mock-treated TSA cells. WGS revealed that TEX DNA cargo represents the entire genome, regardless of RT. More than 99% of TEX DNA was of nuclear origin, but mitochondrial DNA was increased in RT-TEX. Interestingly, we found that RT decreases the level of methylation in both exosomal and total DNA in TSA cells compared to the controls.

**Conclusions:** Our findings support the hypothesis that immunogenic RT dose alters the molecular characteristics of the exosomal DNA cargo, resembling molecular changes occurring in irradiated TEX-producing breast cancer cells. The enrichment in DNA fragments with size of 60–250 bp in RT-TEX is interesting considering that cGAS is fully activated by DNA in this length range [5]. We are currently investigating which features of the cargo DNA that differ between UT-TEX and RT-TEX may explain the differential ability to induce IFN-I pathway activation in recipient DCs. The identification of a DNA signature associated with the ability of TEX to activate the cGAS pathway could provide a circulating biomarker of the RT-driven immunogenic tumor response.

**References**

1. Vanpouille-Box C, Alard A, Aryankalayil MJ, Sarfraz Y, Diamond JM, Schneider RJ, et al. DNA exonuclease Trex1 regulates radiotherapy-induced tumour immunogenicity. Nat Commun. 2017;8:15,618.
2. Mackenzie KJ, Carroll P, Martin CA, Murina O, Fluteau A, Simpson DJ, et al. cGAS surveillance of micronuclei links genome instability to innate immunity. Nature. 2017;548(7668):461–5.
3. Harding SM, Benci JL, Irianto J, Discher DE, Minn AJ, Greenberg RA. Mitotic progression following DNA damage enables pattern recognition within micronuclei. Nature. 2017;548(7668):466–70.
4. Diamond JM, Vanpouille-Box C, Spada S, Rudqvist NP, Chapman JR, Ueberheide BM, et al. Exosomes Shuttle TREX1-Sensitive IFN-Stimulatory dsDNA from Irradiated Cancer Cells to DCs. Cancer Immunol Res. 2018;6(8):910–20.
5. Du M, Chen ZJ. DNA-induced liquid phase condensation of cGAS activates innate immune signaling. Science. 2018;361(6403):704–9.

**Fig. 1:**
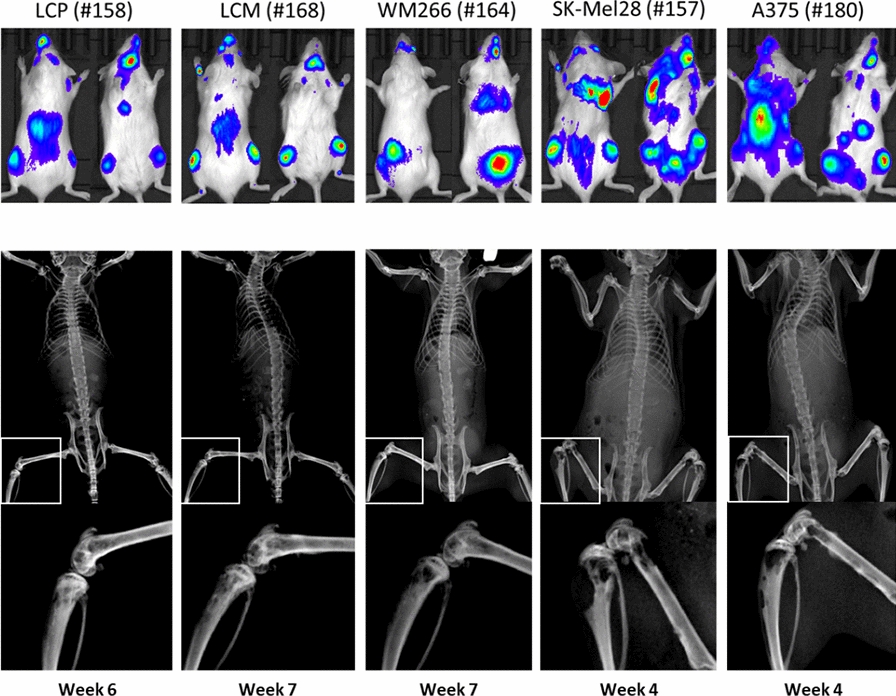
(Abstract 13) The CTA-AT cell line shows typical melanoma phenotype (**a**), an intermediate EMT phenotype (**b**–**d**) and molecules associated with immune escape (**c**)

**Fig. 2:**
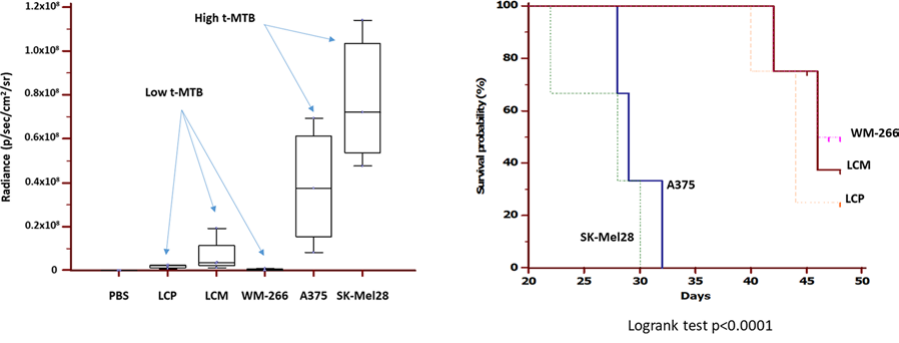
(Abstract 13) The CTC-AT line shows molecular and functional features of cancer stem cells (CSC)

**Fig. 3:**
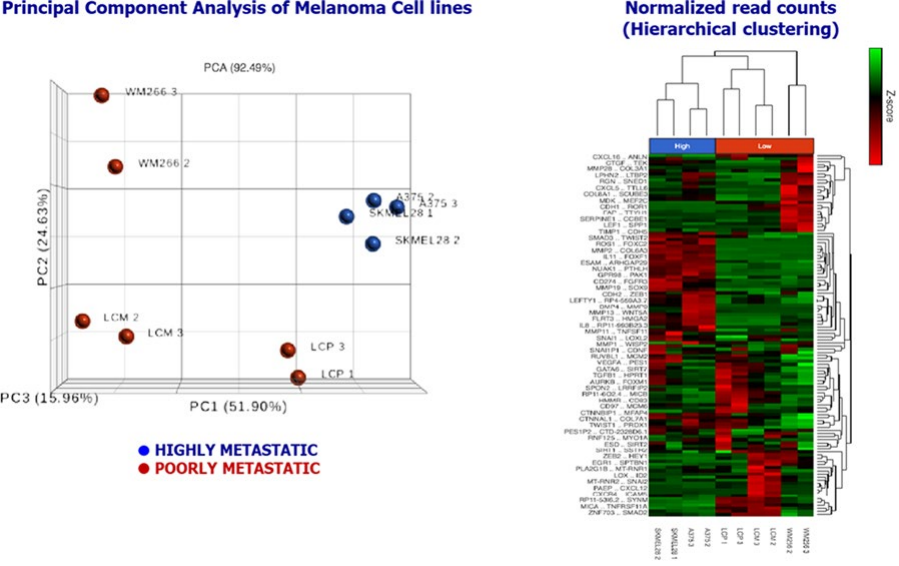
(Abstract 13) The three clonal subpopulations are characterized by different proliferation rate

**Fig. 4:**
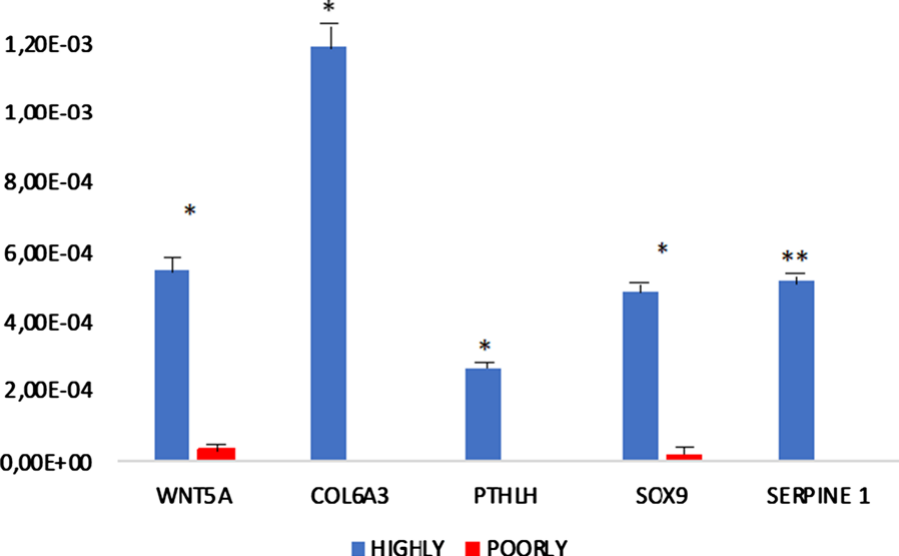
(Abstract 13) The in vivo bioluminescence images display the tumorigenic potential of each clone (**a** and** b**) and the expression of typical melanoma markers by CTC-AT induced metastasis with respect to the primary tissue (**c**)

## 13. The clonal heterogenicity of circulating tumor cells (CTCs) drives their metastatic potential: a new NOD-SCID melanoma model

### Claudia Felici^1^, Paola Cafforio^1^, Francesco Mannavola^1^, Stucci Luigia Stefania^1^, Camillo Porta^1^, Marco Tucci*^1^

#### ^1^Department of Biomedical Sciences and Human Oncology, University of Bari Aldo Moro, Bari, Italy

##### **Correspondence:** Marco Tucci - marco.tucci@uniba.it

*Journal of Translational Medicine* 2020; **19(Supp 1):** 13.

**Background:** Innovative therapies have improved the overall survival in melanoma although a high number of patients still experience disease progression or recurrence. The clonal heterogenicity of melanoma cells is a critical issue concurring to drug-resistance development and metastatic spreading that, however, could be efficiently forecasted by CTC measurement. A number of studies attempted to validate a method for capturing viable CTCs in metastatic melanoma and a peculiar phenotype and biological properties have been recently correlated with outcome.

**Materials and methods:** CTCs were obtained from 15 patients with BRAFV600-mutated metastatic melanoma by DEPArray technology and cultured in vitro. Cells established in vitro were definitely transduced with luciferase. The mutational state was assessed by NGS. The CTC phenotype and molecular profile were investigated by cytometry and qPCR. Stemness was investigated by measuring sphere formation and ALDH activity, while limit dilution discriminated the existence of clonal subpopulations. Viability, proliferation and cell cycle were investigated. The tumorigenic potential of subclones was evaluated by their injection in 8-weeks old NOD/SCID mice. Mice were sacrificed while site of metastasis and tumor burden were evaluated by Lumina-SIII. Metastatic tissues were analysed by immunohistochemistry.

**Results:** A single cell line was established in vitro from a patient with the highest number (n = 102) of CTCs (CTC-AT). Missense mutations of BRAF, TP53 and PIK3CA occurred in CTC-AT and primary tissue. An intermediate EMT phenotype was demonstrated as result of the SNAI1, TWIST1, ZEB1 and ZEB2 or both PDL-1 and CD155 levels (Fig. 1). The expression of CD44, CD90, CD10, CD73 or Oct3/4, Nanog and Sox2 confirmed the presence of stem cells, whereas the ALDH activity and sphere formation suggested the stemness (Fig. 2). Three clonal subpopulations showing different proliferative rate were obtained (Fig. 3). In vivo studies revealed the ability of two clones to develop the tumor, although they showed different metastatic potential (Fig. 4).

**Conclusions:** Herein we described a new NOD-SCID model of metastatic melanoma induced by CTCs. The heterogeneity of subclones as well as their stemness support the role of CTCs for investigating the variability of melanoma behaviour and offers the opportunity of pharmacogenomic studies to guide future therapeutic strategies in advanced disease.

**Fig. 1:**
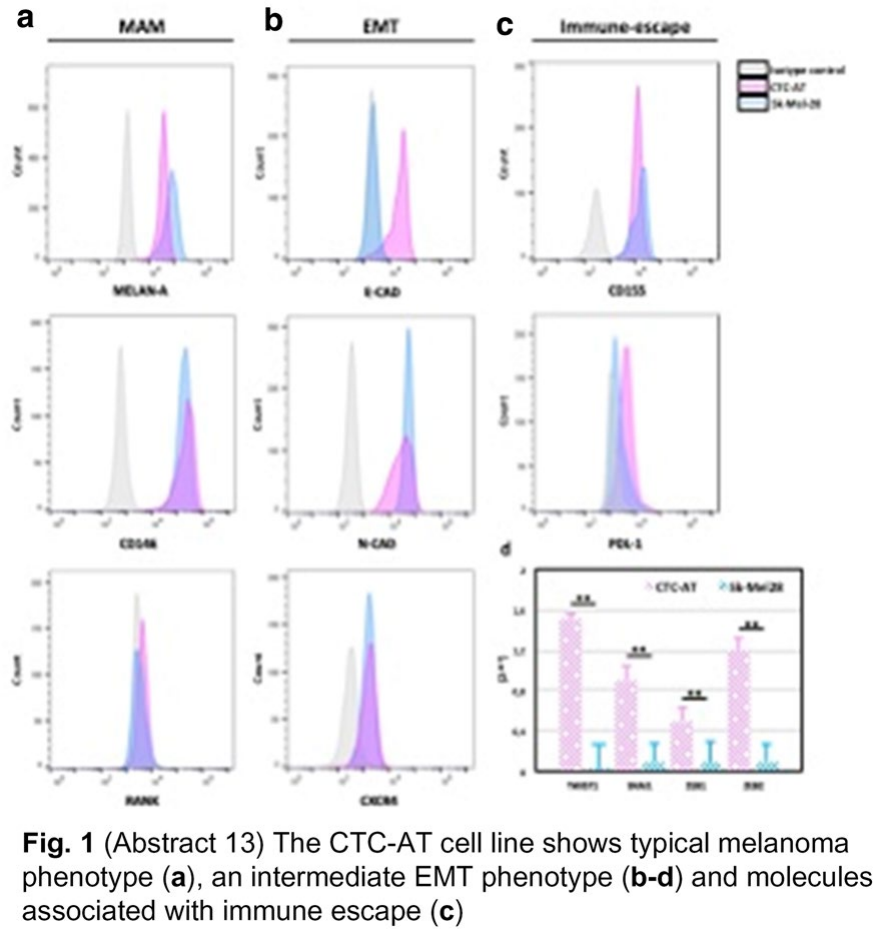
(Abstract 14) Metastatic activity at different time points from intracardiac injection of five melanoma cell lines by in vivo bioluminescence *(upper panel)* and x-ray imaging *(lower panel)*. This Figure 1 belongs to Abstract 14

**Fig. 2:**
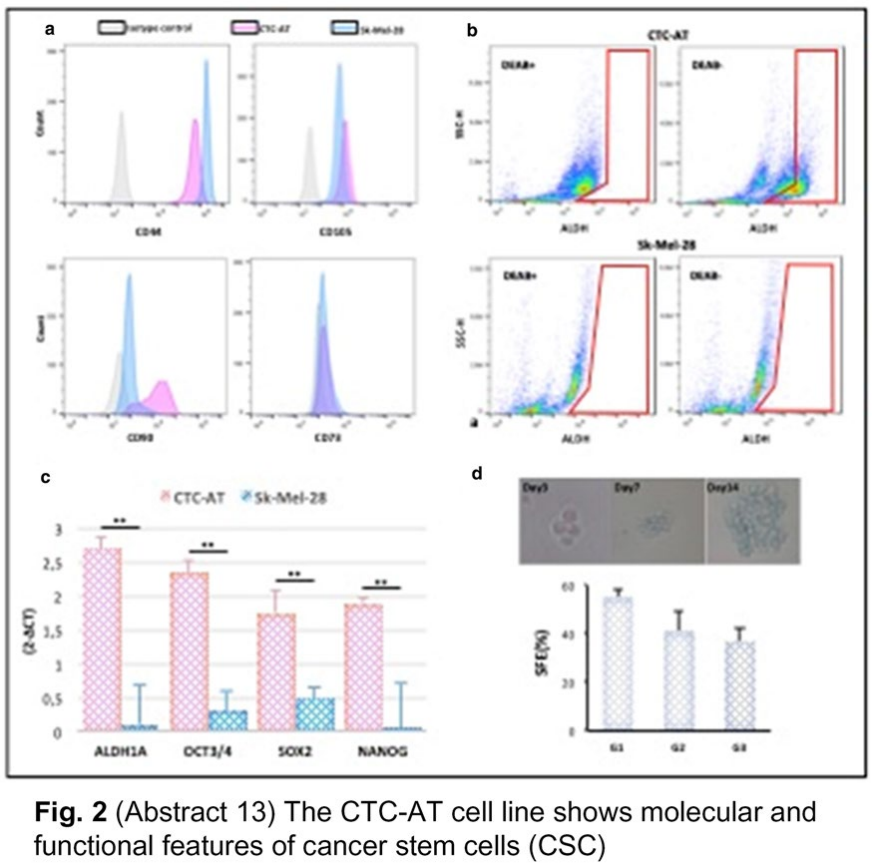
(Abstract 14) Metastatic activity *(left)* and impact on mice survival *(right)* measured by total metastatic tumor burden (t-MTB) and Kaplan-Meyer’s curves. This Figure 2 belongs to Abstract 14

**Fig. 3:**
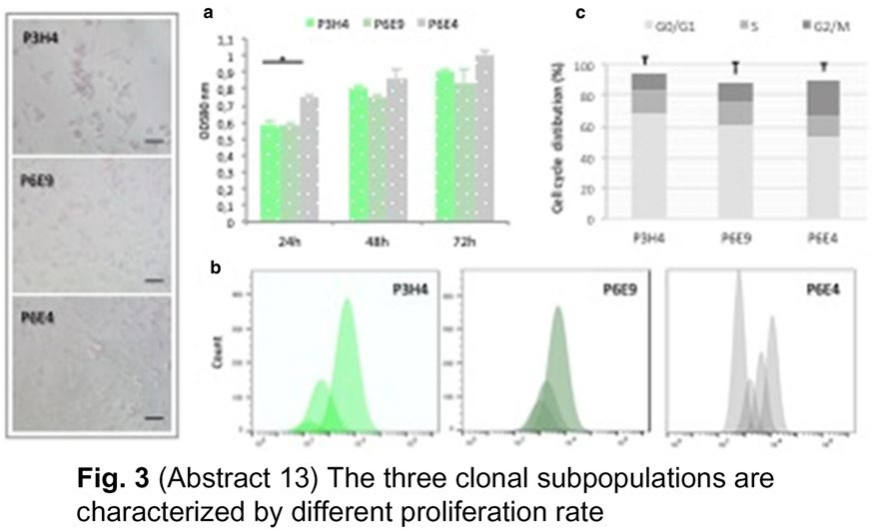
(Abstract 14) Three-dimensional PCA plot (*left*) and 118 genes heatmap with “unsupervised hierarchical clustering” (*right*) by RNAseq of melanoma cell line. This Figure 3 belongs to Abstract 14

**Fig. 4:**
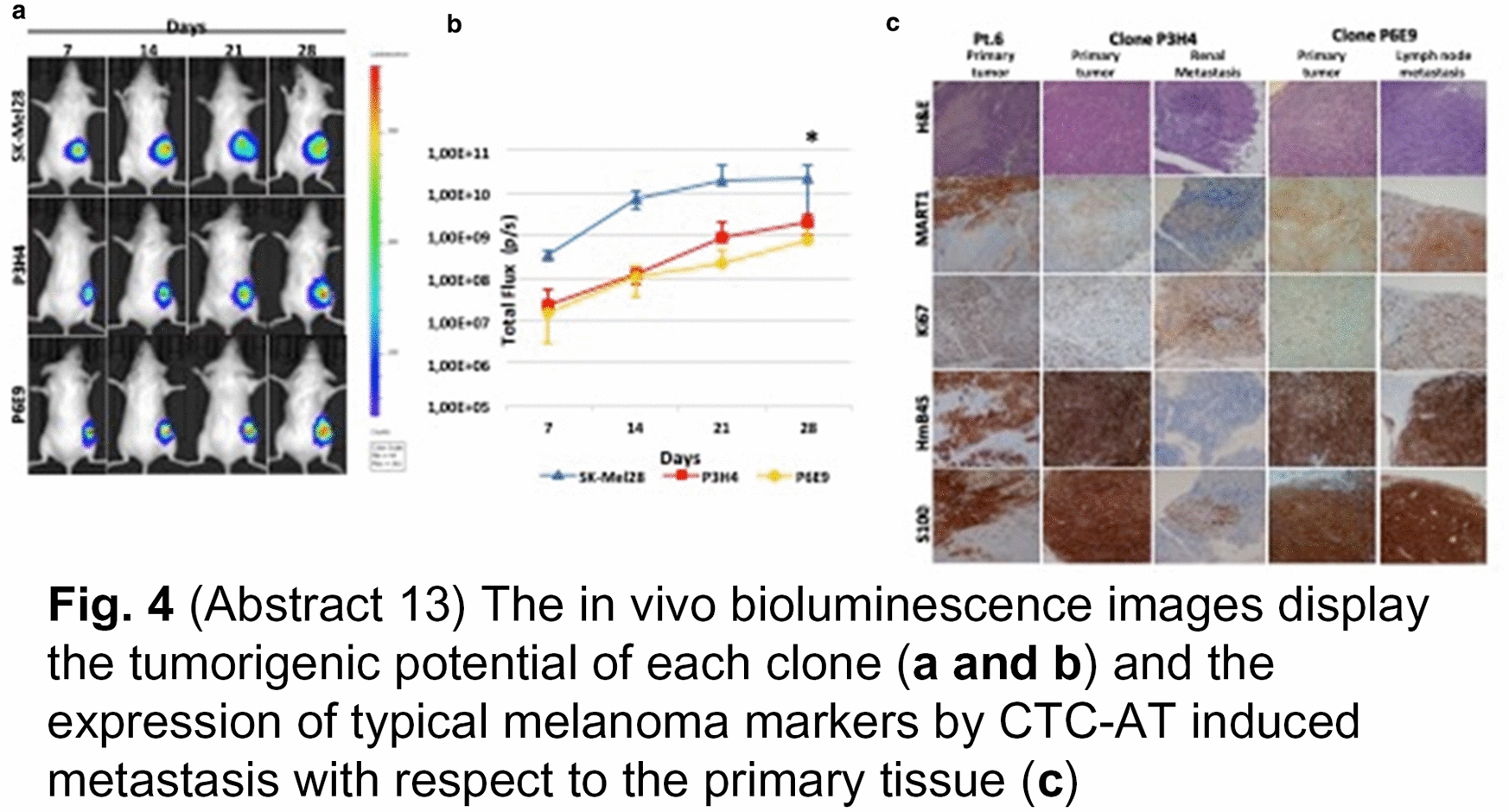
(Abstract 14) Gene expression analysis on FFPE metastatic samples from highly (Group A) *vs* poorly (Group B) metastatic cell lines. This Figure 4 belongs to Abstract 14

## 14. Characterization of the metastatic behaviour and gene expression profile (RNAseq) of different melanoma cell lines: a comprehensive in vivo model.

### Francesco Mannavola^1^, Domenica Lovero^1^, Claudia Felici^1^, Gerardo Cazzato^2^, Marco Moschetta^3^, Mauro Mastropasqua^2^, Camillo Porta^1^ and Marco Tucci*^1^

#### ^1^Section of Medical Oncology, Department of Biomedical Sciences and Clinical Oncology (DIMO), University of Bari ‘Aldo Moro’, Bari, Italy; ^2^Section of Pathology, Department of Emergency and Organ Transplantation (DETO), University of Bari ‘Aldo Moro’, Bari, Italy; ^3^Breast Care Unit, Department of Emergency and Organ Transplantation (DETO), University of Bari Medical School, Bari, Italy

##### **Correspondence:** Marco Tucci - marco.tucci@uniba.it

*Journal of Translational Medicine* 2020; **19(Supp 1):** 14.

**Background:** Metastasis is the major cause of death in malignant melanoma. Several factors, including clinical-pathological and tumor biological features may restrain prognosis. Molecular mechanisms regulating melanoma progression and metastasis have been partially discovered, and thus we developed an in vivo model of metastatic melanoma to investigate potential genes implicated in these events.

**Materials and methods:** To evaluate the in vivo metastatic activity of five different melanoma cell lines (LCP, LCM, WM266, SK-Mel28 and A375), we completed intra-cardiac (ic) injection of 1 × 10^6^ luminescent cells or PBS as control in three NOD-SCID mice *per* cell line. After 3 weeks, mice were studies by in vivo bioluminescence imaging (IVIS Lumina LT) to measure the mean radiance (p/sec/cm^2^/sr), that we arbitrarily considered as a surrogate of the total metastatic tumor burden (t-MTB). Animals were euthanized at the humane endpoints achievement and underwent X-ray evaluation for bone metastasis detection. The Kaplan-Meyer curves described the time to sacrifice from ic injection. Mouse necropsy identified metastatic organs that were explanted and processed for both histology and gene expression analysis. Melanoma cell lines were profiled for gene expression (RNAseq) of 118 genes notably involved in cancer progression and metastasis. Quantitative RT-PCR (qRT-PCR) explored the expression of a restrict number of genes on formalin-fixed paraffin-embedded (FFPE) metastatic samples from euthanized animals.

**Results:** All melanoma cell lines demonstrated a metastatic behaviour following ic injection (Fig. 1) with a variable attitude to produce bone and visceral metastasis (Table 1). Mice injected with LCP, LCM and WM266 showed a lower t-MTB and increased survival (Fig. 2) as compared to A375 and SK-Mel28. Thus, we arbitrarily defined LCP, LCM and WM-266 cell lines ‘poorly metastatic’ (group A), while A375 and SK-Mel28 ‘highly metastatic’ (group B).

The *principal component analysis* and the *unsupervised hierarchical clustering* (118 gene transcriptome heatmap) revealed similar gene expression profiling among cell lines grouped for the metastatic attitude (Fig. 3). The gene expression analysis (Fig. 4) performed on FFPE samples identified five deregulated genes (*WNT5A, COL6A3, PTHLH, SOX9* and *SERPINE1*) between A and B.

**Conclusions:** We describe the metastatic capacity of five melanoma cell lines. Gene expression profiling revealed the activation of five genes as putatively responsible for the high aggressiveness of A375 and SK-Mel28 cells. These results suggest to investigate these genes in a clinical setting and their possible application as druggable target for future therapeutic strategies.

**Table 1:** (Abstract 14) In vivo organotropism of different melanoma cell lines following intracardiac injection in NOD-SCID mice by x-ray and histology data. This Table 1 belongs to Abstract 14

	Bone	Muscle	Bowel	Adrenal	Lung
LCP	** + + **			** + + **	** + + **^**1**^
LCM	** + + **	** + **		** + + **	** + + **^**1**^
WM166	** + **		** + + **	** + **	** + + **
A375	** + + + **	** + + **			** + **^**1**^
SK-Mel28	** + + + **	** + + **			

^(+)^ number of mice showing specific organ colonization. ^(1)^micrometastasis

## 15. Development of leukoderma as an indicator of clinical response to immune checkpoint inhibitors in melanoma patients

### Sofia Verkhovskaia^1^*, Francesca Romana Di Pietro^1^, Simona Mastroeni^2^, Maria Luigia Carbone^3^, Damiano Abeni^2^, Roberto Morese^1^, Francesca Maria Morelli^1^, Gian Carlo Antonini Cappellini^1^, Stefania D’Atri^4^, Paolo Marchetti^5,6^, Federica De Galitiis^1^, Cristina Maria Failla^3^ and Cristina Fortes^2^

#### ^1^Department of Oncology and Dermatological Oncology, IDI-IRCCS, Rome, Italy; ^2^Epidemiology Unit, IDI-IRCCS, Rome, Italy; ^3^Laboratory of Experimental Immunology, IDI-IRCCS, Rome, Italy; ^4^Laboratory of Molecular Oncology, IDI-IRCCS, Rome, Italy; ^5^Department of Clinical and Molecular Medicine, Oncology Unit, Sant'Andrea Hospital, Sapienza University, Rome, Italy; ^6^Medical Oncology Unit B, Policlinico Umberto I, Sapienza University, Rome, Italy

##### **Correspondence:** Sofia Verkhovskaia - sofiglia@gmail.com

*Journal of Translational Medicine* 2020; **19(Supp 1):** 15.

**Background:** Although the development of immune checkpoint inhibitors (ICI) has revolutionized the treatment of metastatic melanoma in the last years, more than half of treated patients experience disease progression during therapy. Leukoderma is described as a skin depigmentation like the autoimmune cutaneous disease vitiligo. Cases of spontaneous leukoderma have been reported in melanoma patients and are associated with a favorable outcome [1]. Moreover, leukoderma sometime appears in melanoma patients subjected to immunotherapies such as interleukin 2, tumor vaccines or ICI [1, 2]. However, no consensus has been reached regarding the development of leukoderma and improved overall survival. The aim of our study was to evaluate a possible association between the appearance of leukoderma during ICI treatment and a better prognosis.

**Materials and methods:** We identified 280 patients followed at the IDI-IRCCS in the last 10 years who had inoperable or metastatic melanoma and had undergone ICI therapy in any line of treatment. Primary endpoint was to evaluate the association between leukoderma appearance and the risk of mortality. Secondary endpoints were to evaluate the association between leukoderma appearance and the response to treatment or time to the next treatment (TTNT), defined as the time between the start of treatment with ICI and the decision to change the treatment because of no clinical benefit or toxicity.

**Results:** In the multivariate Cox model, a protective effect for mortality was found for the onset of leukoderma (HR = 0.23; 95% CI = 0.11–0.44, p < 0.0001), while high lactate dehydrogenases levels above *upper limits of* *normal* (HR = 2.33; 95% CI = 1.51–3.60, p < 0.0001) and stage M1d (M1d *versus* MIa-M1b) (HR = 2.25; 95% CI = 1.39–3.63, p = 0.001) were associated with an increased risk of mortality. In a sub-group analysis (only cutaneous melanoma in first line treatment, N = 153), occurrence of leukoderma was also an independent predictor factor for duration of clinical benefits measured by TTNT.

**Conclusions:** In conclusion, our findings suggest that leukoderma development could be considered as a biomarker of successful response to ICI treatment.

**Ethics Approval:** For this study, approval was obtained from the Ethics Committee of the IDI-IRCCS (Opinion Register No. 510 and subsequent amendments).

**References**

1. Failla CM, Carbone ML, Fortes C, Pagnanelli G, D’Atri S. Melanoma and Vitiligo: In Good Company. Int J Mol Sci. 2019; 20:5731.
2. Cortellini A, Buti S, Agostinelli V, Bersanelli M. A systematic review on the emerging association between the occurrence of immune-related adverse events and clinical outcomes with checkpoint inhibitors in advanced cancer patients. Semin Oncol. 2019; 46:362–371.

## 16. Response for combination of PV-10 autolytic immunotherapy and immune checkpoint blockade in checkpoint-refractory patients

### Jonathan S Zager ^1^, Amod A Sarnaik ^1^, Shari Pilon-Thomas ^1^, Matthew Beatty ^1^, Dale Han ^2^, Gary Lu ^3^, Sanjiv S Agarwala ^4^, Merrick I Ross ^5^, Keisuki Shirai ^6^, Richard Essner ^7^, Bernard M Smithers ^8^, Victoria Atkinson ^8^, Dominic Rodrigues ^9^, and Eric A Wachter ^9^*

#### ^1^Moffitt Cancer Center, Tampa, FL USA; ^2^Oregon Health & Science University, Portland, OR USA; ^3^ St. Luke’s University Health Network, Bethlehem, PA USA; ^4^Temple University, Philadelphia, PA USA; ^5^MD Anderson Cancer Center, Houston, TX USA; ^6^Dartmouth-Hitchcock Medical Center, Lebanon, NH USA; ^7^John Wayne Cancer Institute, Santa Monica, CA USA; ^8^Princess Alexandra Hospital, Brisbane, QLD, AUS; ^9^Provectus Biopharmaceuticals, Inc., Knoxville, TN USA

##### **Correspondence:** Eric A. Wachter - wachter@pvct.com

*Journal of Translational Medicine* 2020; **19(Supp 1):** 16.

**Background:** PV-10, an injectable formulation of rose bengal disodium, is a small molecule autolytic immunotherapy in development for solid tumors; intralesional injection can yield immunogenic cell death and tumor-specific reactivity in circulating T cells [1–4]. PV-10 is currently the subject of a Phase 1b/2 study in combination with immune checkpoint blockade (CB) in patients with advanced cutaneous melanoma (NCT02557321).

**Materials and methods:** Participants must have at least 1 injectable lesion, at least 1 measurable target lesion (TL), and be candidates for pembrolizumab. Patients receive combination treatment q3w for 5 cycles followed by pembrolizumab alone q3w (total duration of up to 24 months); the primary endpoint is safety and tolerability, with objective response rate (ORR) and progression-free survival (PFS) as key secondary endpoints (assessed via RECIST 1.1 after 15 weeks, then q12w). Immune correlative assessments are being performed on a subgroup of patients.

**Results:** An expansion cohort is accruing up to 24 CB-refractory patients; early results of the first 15 patients (2 Stage M0, 4 M1a, 2 M1b, 5 M1c, 2 M1d) are presented here; all had one or more prior lines of CB (9 were refractory to CTLA-4 and PD-1). Treatment-Emergent Adverse Events were consistent with established patterns, principally Grade 1–2 injection site reactions attributed to PV-10 and Grade 1–3 immune-mediated reactions attributed to pembrolizumab, with no significant overlap or unexpected toxicities. This profile is similar to CB-naïve patients in the main cohort of the study [5]. As of the data cutoff, 12 patients were evaluable for overall response by RECIST: 33% ORR, 1 CR (M1a) and 3 PRs (2 M0 and M1d); 3 patients (M1a, M1b, and M1c) achieved SD for a disease control rate (DCR) of 58%. These patients had 31 TLs; 17 were injected with PV-10 and achieved 29% CR, 41% ORR, and 59% DCR on a per lesion basis. These patterns parallel those observed in CI-naïve patients [5]. Five patients completed correlative assessment: 2 exhibited increased High Mobility Group Box 1 (HMGB1), a DAMP molecule associated with the activation of dendritic cells; and 1 patient refractory to combination CTLA-4/PD-1 exhibited increased T cell reactivity to HLA-matched tumor that preceded a durable CR. Similar immune upregulation has been shown with single-agent PV-10 in CB-naïve patients [4].

**Conclusion:** Encouraging response, both at patient- and injected lesion-levels, coupled with a non-overlapping safety profile, support ongoing enrollment. Pharmacodynamic assessments substantiate PV-10′s immune-mediated mechanism in CB-refractory patients.

**References**

1. Wachter E, Dees C, Harkins J, Fisher W, Scott T. Functional imaging of photosensitizers using multiphoton microscopy. Proceedings of SPIE, Multiphoton Microscopy in the Biomedical Sciences II, Periasamy, A. and So, P.T.C. (eds), Bellingham, Washington: 2002; 4620:143–147.
2. Liu H, Innamarato PP, Kodumudi K, Weber A et al. Intralesional rose bengal in melanoma elicits tumor immunity via activation of dendritic cells by the release of high mobility group box 1. Oncotarget 2016; 7:37893-37905.
3. Qin J, Kunda N, Qiao G, Calata JF et al. Colon cancer cell treatment with rose bengal generates a protective immune response via immunogenic cell death. Cell Death and Disease 2017; 8:e2584, Doi: https://doi.org/10.1038/cddis.2016.473.
4. Liu H, Weber A, Morse J, Kodumudi K et al. T cell mediated immunity after combination therapy with intralesional PV-10 and blockade of the PD-1/PD-L1 pathway in a murine melanoma model. PLoS One 2018; 13:e0196033. https://doi.org/10.1371/journal.pone.0196033.
5. Agarwala S, Ross M, Zager J, Sarnaik A et al., A phase 1b study of rose bengal disodium and anti-PD-1 in metastatic melanoma: results in patients naïve to immune checkpoint blockade. ESMO Virtual Congress 2020, presentation 1125P.

## 17. The impact of brain involvement in metastatic melanoma patients treated with pembrolizumab

### Michael Weichenthal^1,2^*, Scherrer Emilie^3^, Sheenu Chandwani^3^, Peter Mohr ^4,5^ Ulrike Leiter^6^, Selma Ugurel^7^, Katharina C. Kähler^1,8^, Ralf Gutzmer^9^, Claudia Pföhler^10^, Jessica Hassel^11^, Patrick Terheyden^12^, Beatrice Schell^13^, Jochen Utikal^14^: Alexander Kreuter^15^, Sebastian Haferkamp^16^, Anca Sindrilaru^17^, Jessica Hassel^18^, Dorothee Nashan^19^, Kjell M. Kaune^20^, Carola Berking^21^, Dirk Debus^22^, Jens Ulrich^23^, Evelyn Dabrowski^24^, Thomas Eigentler^6^, Rudolf Herbst^25^, Julia Welzel^26^, Carmen Loquai^27^, Friedegund Meier^28^, Dirk Schadendorf^7^

#### ^1^Department of Dermatology, University of Kiel, Kiel, Germany; ^2^The ADOReg Study Group, Berlin, Germany; ^3^ Merck & Co., Inc., Kenilworth, NJ, USA; ^4^Elbe-Kliniken Buxtehude, Buxtehude, Germany; ^5^Skin Cancer Center, Buxtehude, Germany; ^6^Skin Cancer Center, Tübingen, Germany; ^7^University of Essen, Essen, Germany; ^8^Skin Cancer Center, Kiel, Germany; ^9^Skin Cancer Center, Hannover, Germany; ^10^Saarland University Hospital, Homburg, Germany; ^11^University of Heidelberg, Heidelberg, Germany; ^12^Skin Cancer Center, Lübeck, Germany; ^13^Department of Dermatology, Gera, Germany; ^14^University Department of Dermatology, Mannheim, Germany; ^15^St. Elisabeth Hospital, Oberhausen, Germany; ^16^University Department of Dermatology, Regensburg, Germany; ^17^University Department of Dermatology, Ulm, Germany; ^18^University Department of Dermatology, Heidelberg, Germany; ^19^Skin Cancer Center, Dortmund, Germany; ^20^Skin Cancer Center, Bremen, Germany; ^21^University Department of Dermatology, Munich, Germany; ^22^Skin Cancer Center, Nuremberg, Germany; ^23^Skin Cancer Center, Quedlinburg, Germany; ^24^Skin Cancer Center, Ludwigshafen, Germany; ^25^Skin Cancer Center, Erfurt, Germany; ^26^University of Augsburg, Augsburg, Germany; ^27^Skin Cancer Center, Mainz, Germany; ^28^University Department of Dermatology, Dresden, Germany

##### **Correspondence:** Michael Weichenthal - mweichenthal@dermatology.uni-kiel.de

*Journal of Translational Medicine* 2020; **19(Supp 1):** 17.

**Background: **The anti-PD1 antibody pembrolizumab is established as a standard of care in European patients with metastatic melanoma (MM). Melanoma brain metastases (MBM) are a common and serious tumour manifestation in MM. We examined pembrolizumab use and associated outcomes in patients with MBM.

**Materials and methods:** Real-world data from the German ADOReg skin cancer registry was used to evaluate 664 adult patients with advanced stage IV or non-resectable stage III melanoma on pembrolizumab treatment with follow-up through December 31, 2019. Overall survival (OS) and real-world time on treatment (rwToT) from pembrolizumab initiation were analysed for patients with and without known baseline MBM using the Kaplan-Meier (KM) method. Multivariable Cox regression was used to control for potential confounding factors including performance status (ECOG), line of treatment, serum lactate dehydrogenase (LDH), and number of metastatic sites. For patients with MBM, the number of brain lesions and the role of concurrent radiotherapy were analysed.

**Results: **Pembrolizumab was administered to 401 (60%), 143 (22%), and 120 (18%) patients as first, second-, or higher line of treatment, respectively. Overall, 39% had elevated LDH, and 27% had ECOG ≥1. Most patients (66.4%) had two or more metastatic sites, and 164 (24.7%) patients had brain metastases at pembrolizumab initiation

Survival at 24 months was similar between patients with a single brain lesion (58.7%) and those without brain involvement (59%), but lower for patients with multiple MBM (40.0%). (Table 1). Patients without known baseline MBM had a median OS of 32.8 (95% CI 27.6–39.8) months, while those with a single or multiple brain lesions had median OS of 47.2 (95% CI 16.00-NR) months and 19.6 (95%CI 13.7–30.3) months, respectively (p<0.01).

KM median rwToT was similar for patients with (6.0 months) or without MBM (6.3 months). (Table 1) The observed difference between patients with one brain lesion (6.5 months) and those with multiple MBM (5.1 months) was not significant (p=0.23).

Radiotherapy to the brain was delivered upfront or concurrently with pembrolizumab in similar rates for patients with single brain lesions (32.6%) and multiple lesions (34.9%). Concurrent radiotherapy did not significantly affect OS or rwToT in patients with MBM in multivariable Cox analysis.

**Conclusion: **These findings support effectiveness of pembrolizumab in real-world clinical settings and in patients with MBM. Patients with a single brain lesion and those without MBM seem to benefit equally from pembrolizumab treatment. Previously published findings suggesting a synergistic effect of concurrent brain radiotherapy could not be confirmed.

**Table 1:** (Abstract 17) Summary of real-world outcomes of patients with melanoma brain metastasis in the German ADOReg skin cancer registry. This Table 1 belongs to Abstract 17

	No melanoma brain metastases N = 500	Any melanoma brain metastases		
		At least one melanoma brain metastasis N = 164	Single melanoma brain metastasis N = 46	Two or more melanoma brain metastases N = 106
Survival rate at 24 months, %	58.9%	45.7%	58.7%	40.0%
Median OS in months (95% CI)	32.8 (27.6–39.8)	19.6 (13.7—30.3)	47.2 (16.0—NR)	16.7 (10.4 – 25.1)
Median rwToT in months (95% CI)	6.3 (5.3 – 7.2)	6.0 (4.1 – 6.9)	6.5 (3.1 – 12.5)	5.1 (3–5 7.2)

*12 patients had brain metastasis information at baseline, but the number of lesions was unknown; these patients were excluded from analysis by number of brain lesions

